# Effects of aerobic and combined training on pain-free walking distance and health-related quality of life in patients with peripheral artery disease: a randomized clinical trial

**DOI:** 10.1590/1677-5449.202300242

**Published:** 2023-08-28

**Authors:** Eduardo Lima Garcia, Adamastor Humberto Pereira, Marcio Garcia Menezes, Alexandre Araújo Pereira, Ricardo Stein, Leandro Tolfo Franzoni, Luiz Claudio Danzmann, Antônio Cardoso dos Santos

**Affiliations:** 1 Universidade Federal do Rio Grande do Sul - UFRGS, Porto Alegre, RS, Brasil.; 2 Hospital de Clínicas de Porto Alegre - HCPA, Porto Alegre, RS, Brasil.; 3 Hospital Moinhos de Vento, Porto Alegre, RS, Brasil.; 4 Universidade Luterana do Brasil, Canoas, RS, Brasil.

**Keywords:** peripheral artery disease, pain-free walking distance, health-related quality of life, exercise, intermittent claudication, doença arterial periférica, distância percorrida livre de dor, qualidade de vida relacionada à saúde, exercício, claudicação intermitente

## Abstract

**Background:**

Decreased walking ability in patients with peripheral arterial disease is often a clinical problem and limits the quality of life and daily activities of these subjects. physical exercise is important in this scenario, as it improves both the daily walking distance and the ability to withstand intermittent claudication related to the limitations of the peripheral disease.

**Objectives:**

Our aim was to compare the effects of two types of exercise training (aerobic training and aerobic training combined with resistance exercises) on pain-free walking distance (PFWD) and health-related quality of life (HRQoL) in a sample composed of patients with peripheral artery disease (PAD).

**Methods:**

Twenty patients with claudication symptoms were randomized to either aerobic control (AC) **N= 9,** or combined training (CT) N= 8, (24 sixty-minute sessions, twice a week). The total walking distance until onset of pain due to claudication was assessed using the 6-minute walk test and HRQoL was measured using the WHOQOL-bref questionnaire (general and specific domains) at baseline and after training. We used generalized estimating equations (GEE) to assess the differences between groups for the PFWD and HRQoL domains, testing the main group and time effects and their respective interaction effects. P values < 0.05 were considered statistically significant.

**Results:**

Seventeen patients (mean age 63±9 years; 53% male) completed the study. Both groups experienced improvement in claudication, as reflected by a significant increase in PFWD: AC, 149 m to 299 m (P<0.001); CT, 156 m to 253 m (P<0.001). HRQoL domains also improved similarly in both groups (physical capacity, psychological aspects, and self-reported quality of life; P=0.001, P=0.003, and P=0.011 respectively).

**Conclusions:**

Both aerobic and combined training similarly improved PFWD and HRQoL in PAD patients. There are no advantages in adding strength training to conventional aerobic training. This study does not support the conclusion that combined training is a good strategy for these patients when compared with classic training.

## Resumo

**Contexto:**

A diminuição da capacidade de marcha em pacientes com doença arterial periférica é frequentemente um problema clínico e limita a qualidade de vida e as atividades diárias desses indivíduos. O exercício físico é importante nesse cenário, pois melhora tanto a distância caminhada diária quanto a capacidade de suportar a claudicação intermitente relacionada às limitações da doença periférica.

**Objetivos:**

Comparar os efeitos do treinamento aeróbico (TA) e do treinamento aeróbico combinado com exercícios de resistência (TC) na distância percorrida livre de dor (DPLD) e na qualidade de vida relacionada à saúde (QVRS) em pacientes com doença arterial periférica (DAP).

**Métodos:**

Vinte pacientes com sintomas de claudicação foram randomizados para TA ou TC. Os treinamentos foram realizados em 24 sessões, duas vezes por semana. A DPLD foi avaliada por meio do teste de caminhada de 6 minutos, e a QVRS foi medida pelo instrumento da avaliação de qualidade de vida da Organização Mundial da Saúde (WHOQOL-BREF), no início e após o treinamento. Para avaliar as diferenças entre os grupos para DPLD e os domínios da QVRS, foi utilizado o modelo de equações de estimativa generalizada, testando os efeitos principais do grupo e tempo, bem como os respectivos efeitos de interação. Valores de p < 0,05 foram considerados estatisticamente significativos.

**Resultados:**

Dezessete pacientes (idade média: 63±9 anos; 53% do sexo masculino) completaram o estudo. Ambos os grupos apresentaram melhora na claudicação, refletida por um aumento significativo na DPLD: grupo controle aeróbico - de 149 m para 299 m (P < 0,001); grupo de treinamento combinado - de 156 m para 253 m (P < 0,001). Os domínios da QVRS também melhoraram de forma semelhante em ambos os grupos (capacidade física, aspectos psicológicos e qualidade de vida autorreferida; P = 0,001, P = 0,003 e P = 0,011, respectivamente).

**Conclusões:**

Ambos os treinamentos melhoraram de forma semelhante a DPLD e a QVRS em pacientes com DAP. Não há vantagens em associar o treinamento de força ao treinamento aeróbico convencional. O estudo não permite concluir que o TC é uma boa estratégia para esses pacientes quando comparado ao treinamento clássico.

## INTRODUCTION

Exercise training is key to improving claudication symptoms in patients with peripheral artery disease (PAD).^1^ A wealth of evidence recommends exercise training for prevention and rehabilitation of atherosclerotic disease progression.^2,3^ In this scenario, increasing pain-free walking distance (PFWD) is one of the main goals when treating patients with PAD.^4^ Intermittent claudication (IC) is a particularly relevant factor, as it constitutes a limiting barrier to physical activity and engagement in exercise training.^5^

Both aerobic and combined exercise can be used to improve PFWD, which, in turn, has positive impacts on functional capacity and health-related quality of life (HRQoL).^6^ Guidelines recommend aerobic exercise to improve PFWD, resulting in better peripheral vasodilatation due to nitric oxide release.^7^ On the other hand, combined exercise may improve dynamic stability by increasing leg strength and thus improve PFWD.^8^ A recent systematic review showed that there is insufficient evidence about the effects of combined exercise compared to isolated aerobic exercise.^9^ Therefore, there is no consensus on which exercise modality is superior for improving IC.

In addition to the lack of consensus on which modality is superior, few studies have directly compared these different training modalities in PAD patients and their effects therefore remain unclear. Furthermore, it is unknown whether resistance training can have a negative or countervailing influence on the effects of aerobic exercise, generating concurrent effects and decreasing the potential positive effects on PFWD.^10^

In the present study, we compared the effects of aerobic training or combined training on PFWD, general HRQoL, and specific QoL domains in patients with PAD. We hypothesized that combined training might promote further improvements in PFWD and QoL when compared to aerobic training alone.

## MATERIALS AND METHODS

### Subjects

Patients with IC were recruited from the outpatient vascular surgery clinic of a university hospital in southern Brazil. The inclusion criteria were age over 40 and ankle-brachial index (ABI) below 0.9. The exclusion criteria were cardiovascular events occurring <3 months before inclusion, uncontrolled severe hypertension (systolic blood pressure ≥ 180 mmHg or diastolic blood pressure ≥ 110 mmHg) and/or uncontrolled diabetes (glycemic index ≥ 290), critical limb ischemia, limiting pulmonary disease, and any contraindication to exercise.

### Study design

**Figure 1:**
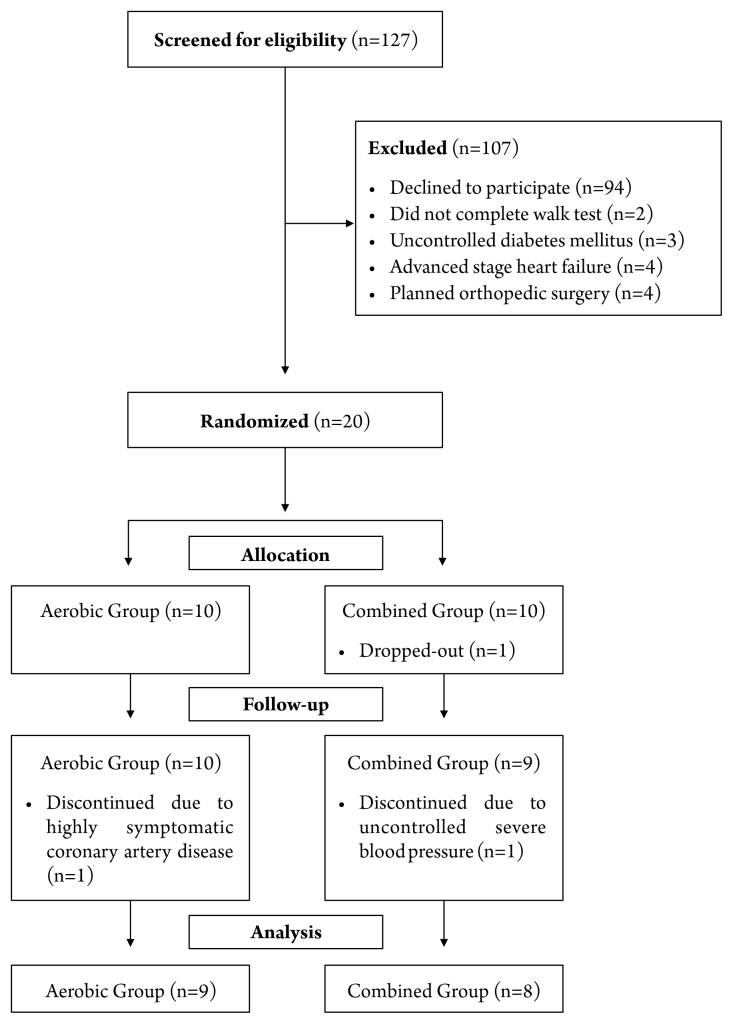
Study Flowchart (CONSORT).

A randomized clinical trial was conducted in accordance with the Consolidated Standards of Reporting Trials (CONSORT) guidelines (Figure1). The research was conducted as an “intention to treat” analysis. Participants were randomized into two groups using an online randomizer and the sealed envelopes method, with randomization concealed from the recruiting investigator. All measurements were performed at baseline and after the intervention program.

### Experimental procedure

#### Walking capacity and pain-free walking distance

Walking capacity was measured with the 6-minute walk test (6MWT) by a team with previous experience, in accordance with American Thoracic Society guidelines.^11^ During the 6MWT, we used a visual analogue scale (VAS) to measure pain in the lower limbs.^12^ The test was conducted along a 30-meter corridor where each patient was asked to walk the longest possible distance in 6 minutes. Blood pressure, heart rate, pain scale, and Borg CR-10 scale of perceived exertion were measured before and after the test.^13^ During the 6MWT, patients were asked about the onset of claudication pain and instructed to walk as briskly as possible. The distance walked until the onset of claudication pain and the total distance walked were recorded and expressed in meters.

#### Health-related quality of life

HRQoL was evaluated using the Portuguese-language version of the World Health Organization Quality of Life questionnaire, short form (WHOQOL-bref).^14^ The WHOQOL-bref is composed of five domains that cover physical capacity, psychological aspects, social relations, environment, and self-reported QoL. The questionnaire consists of 26 items, with the first and second measuring general QoL. Item scores range from 1 to 5. The appropriate syntax was used to calculate scores in each domain. The higher the score, the better the respondent’s QoL. The maximum and minimum possible scores are 20 and 4, respectively.

#### Training programs

The intervention program lasted 12 weeks, with two sessions per week. The aerobic exercise group and combined group (CAG and TCG, respectively) trained for a total of 60 minutes per session. Aerobic training for both groups consisted of 30-minute sessions on a treadmill. Resistance training lasted 20 minutes and was composed of five exercises for the upper and lower limbs. The only difference between CAG and TCG was that the warm-up and cool-down periods had a longer duration, because the combined group (by definition) also performed resistance training.

The CAG intensity was set at around 40-60% of heart rate reserve (HRR), which was calculated using the formula HRR = [(maximum heart rate - resting heart rate) x % intensity] - resting heart rate. To predict maximum heart rate (HRmax), we used the formula HRmax = 208 - (0.7 x age) for patients not on beta blockers and HRmax = 164 - (0.7 x age) for patients on beta blockers.^15^

The TCG performed resistance training with a load of 4 to 7 on the OMNI scale of perceived exertion for resistance exercise.^16^ We used a validated method to prescribe the intensity of resistance training: in the initial training phase, the number of repetitions was 15, with a reduction to 10 as training progressed. Five basic resistance exercises were prescribed: bench press, horizontal elbow flexion (for the back), elbow flexion (for the biceps), knee extension, and plantar flexion.

### Statistical procedures

To calculate sample size, we used a difference of 125 meters (standard deviation 86 meters) in PFWD between groups, with power of 0.8, and a 5% statistical significance level (p<0.05). The sample size was calculated as 8 patients per group. To account for a 20% rate of sample loss and attrition, the final sample size was calculated as 20 patients.^17^

We used Shapiro Wilk and Levene tests to analyze the normality and homogeneity of data, respectively. To analyze between-group differences in sample characteristics, we used a *t* test (unpaired) or the Mann-Whitney *U* test for continuous variables and the chi-square test for categorical variables. These data were expressed as means and standard deviation if distributed normally, or median and interquartile range otherwise.

Generalized Estimating Equations (GEE) and Bonferroni post hoc tests were used to compare the data for all dependent variables (PFWD and HRQoL). So, the factors adopted in this analysis were “group” (CAG and TCG) and “time” (pre and post training period). PFWD data were expressed as means and 95% confidence interval and HRQoL data were expressed as means and standard deviations, all analyzed by intention-to-treat. All data were analyzed in SPSS Version 20.0 (IBM Corporation, Armonk, NY). P*-*values <0.05 were considered statistically significant.

### Ethics

The trial was approved by the local research ethics committee under number 2014-0381, CAAE 37493214.5.0000.5327, and conducted entirely in accordance with the ethical standards set forth in the Declaration of Helsinki. All patients provided written informed consent for participation before enrollment.

This study is registered on the ClinicalTrials.gov platform with accession number NCT02729090.

## RESULTS

Seventeen of the 20 patients initially allocated completed the study. All participants had 100% adherence to the exercise sessions. Sample characteristics at baseline are presented in Table 1. One patient dropped-out before allocation to the TCG due to financial difficulties to pay for transport. During follow-up, one patient from the CAG discontinued due to highly symptomatic coronary artery disease (i.e., angina), reporting symptoms during activities of daily living. Finally, one CAG patient discontinued due to uncontrolled severe blood pressure, monitored during the pre-exercise routine.

**Table 1:** Sample characteristicvs of both training groups.

**Characteristics**	**Aerobic Group (N=9)**	**Combined Group (N=8)**	**P value**
Age (years)	65 ± 3	61 ± 3	0.376
Body mass (kg)	82 ± 4	73 ± 4	0.141
Height (cm)	172 ± 2	165 ± 2	0.006*
Female n (%)	2 (22%)	6 (75%)	0.030*
6MWT (meters)	336 ± 82	366 ± 99	0.726
BMI	28 ± 2	27 ± 2	0.755
ABI	0.78 (0.73; 0.87)	0.74 (0.70; 0.83)	0.326
Fontaine 2b class n (%)	9 (100%)	8 (100%)	--------
Risk Factors			
Hypertension n (%)	9 (100%)	6 (75%)	0.206
Diabetes Mellitus n (%)	5 (56%)	5 (62%)	1.000
Dyslipidemia n (%)	9 (100%)	8 (100%)	--------
Smokers n (%)	3 (33%)	3 (37%)	1.000
Ex-smokers n (%)	1 (11%)	3 (37%)	0.294
Never smoked n (%)	5 (56%)	2 (25%)	0.335
Alcoholism n (%)	2 (22%)	5 (62%)	0.153
Drug therapy			
Anti-hypertensives n (%)	9 (100%)	6 (75%)	0.206
Glycemic control n (%)	5 (56%)	5 (62%)	1,000
Beta-blockers n (%)	6 (67%)	3 (37%)	0.347

* indicates statically significant difference.

BMI: body mass index; ABI: ankle-brachial index; 6MWT: six-minute walk test. *T*-test (unpaired), Mann-Whitney *U* test, and chi-square test were used to indicate statistical difference. Source: the authors.

PFWD did not differ between groups at baseline or at completion of the intervention period (P=0.677). However, both groups demonstrated improvements after 12 weeks of training (P<0.001), without significant interactions (P=0.155). The median baseline value in the CAG was 149 (124-179) m, improving to 299 (249-359) m after the training program. For the TCG, the baseline value was 156 (118-208) m, increasing to 253 (211-303) m after training.

QoL also improved significantly in both groups, specifically in three domains: physical capacity, psychological aspects, and self-reported QoL (P=0.001, P=0.003 and P=0.011, respectively) (Table 2). Again, there was no interaction for group or for group vs. time.

**Table 2:** Mean ± Standard Error of WHOQOL-bref questionnaire domain scores.

	**Aerobic Group (N=9)**		**Combined Group (N=8)**		**P value**
	Pre	Post	Pre	Post	Time
Physical Capacity	11.9 ± 0.8	14 ± 0.7	12.4 ± 0.7	14.6 ± 0.5	0.001
Psychological Aspects	14.1 ± 0.9	15.4 ± 0.7	14.8 ± 0.6	16.4 ± 0.5	0.003
Self-reported QoL	12.2 ± 0.8	15.1 ± 0.6	13.7 ± 1	15 ± 0.7	0.011

Pre: before training period; Post: after training period; P value < 0.05 indicates statistically significant difference. Generalized Estimating Equations (GEE) and Bonferroni post hoc tests were performed to indicate statistical difference. Source: own author.

## DISCUSSION

The main finding in our study was that 12 weeks of low-tech interventions improve PFWD and HRQoL in patients with PAD. In our opinion, this is an important aspect because, beyond the known financial savings and lower complication rate of this low-tech intervention over endovascular intervention,^18^ our results also showed positive effects on claudication symptoms and HRQoL. Training programs for both groups were prescribed with individualized intensities, using the HRR as a basis. The combined training approach included both aerobic and resistance exercises, which seems to be easily applicable for this patient subgroup.

**Figure 2:**
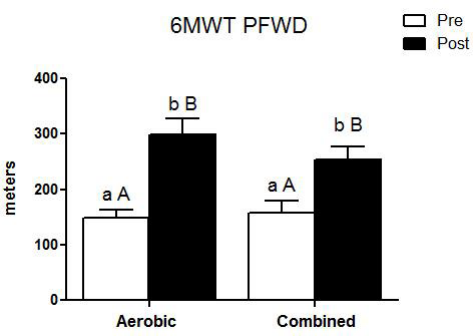
Pain-free walking distance in the Six Minute Walking Test. Lower-case letters indicate statistically significant differences for the same group (pre-intervention compared with post-intervention). Capital letters indicate statistically significant differences between groups for the same time.

The main finding of the present study was that both groups experienced improvements in PFWD and HRQoL, demonstrating that combined training was as effective as aerobic training regarding these relevant outcomes. After the training period, there were significant effects for onset of claudication pain during walking in both groups (Figure 2). Our hypothesis was that combined training might provide greater benefits due to the sum of the effects of aerobic and resistance exercises in the same session. Aerobic training enhances peripheral blood flow, which can lead to increased nitric oxide bioavailability, thus improving peripheral vasodilation.^7^ Additionally, resistance training can strengthen the core and lower limbs, leading to greater walking ability and stability.^19^ However, our hypothesis was not confirmed, because both training groups demonstrated similar improvements in PFWD. Patients in the CAG improved PFWD by ~101% while those in the TCG improved by ~62% after 3 months of exercise training, both relevant results with clinical impact for PAD patients. Similar results were found by McDermott et al.^2^ (~118% for aerobic training, although in 6 months). It is possible that aerobic training alone may have influenced resistance training without provoking any additional benefits for the combined vs. aerobic groups.^10,20^ It is important to point out that aerobic training may limit some of the adaptations of resistance training when the two are performed in the same session, due to inhibition of muscle hypertrophy and reduced testosterone levels.^21^ In contrast, it is still unclear whether aerobic training can interfere negatively with resistance training, making combined training strategies no better than aerobic training to improve PFWD in patients with PAD. Some evidence suggests that combined training compared with aerobic training is more effective for improving functional capacity, with increases of up to 18% in peak oxygen uptake and 38% in lower limb muscle strength, without any untoward interference from the two different exercise modes in the same session.^22^ The key point seems to be the volume and number of exercises used for resistance training. One effective strategy may be to prescribe few, high-intensity repetitions for combined training to have positive effects.^23^

Regarding HRQoL, both groups exhibited significant increases in the physical capacity, psychological aspects, and self-reported QoL domains after the training program and these were directly related to the improvement in PFWD. Healthy individuals without impairment in walking ability have better QoL compared to patients with PAD,^24^ hence the importance of physical exercise in this patient population to improve PFWD and, consequently, QoL.^25,26^ As individuals are able to walk longer distances without feeling pain, their perception of their physical capacity tends to increase.^24,27^ In addition, the psychological domain also improved for both groups, with particular emphasis on a single questionnaire item that addresses the patient’s self-esteem.^28^ When patients can walk without pain for longer, they experience improvements in self-esteem and, consequently, overall improvement in the psychological domain.^29^ Finally, self-reported QoL also improved. In general, the improvement in QoL perceived by patients converges with improvement in the physical and psychological aspects. These results confirm the importance of physical exercise for these patients.^30,31^

Some limitations of the present study must be noted. First, the intervention time was relatively short. For future studies, we suggest a period longer than 12 weeks and three weekly sessions, mainly due to the initial adaptations to resistance training, which are predominantly neural during the first weeks. Second, the dose response of resistance training was small because we did not perform the one-repetition maximum (1RM) test to quantify training load, which may have underestimated exercise intensity, although we did use the OMNI scale to control intensity. Third, the absence of a resistance training only group, or of a control group, may be a limitation, since we do not know whether one method actually interfered with the other. Finally, caution should be exercised when generalizing the results of the present study to the broader population of patients with PAD, because our sample was small and deliberately restricted to patients in a specific functional class (Fontaine 2b). On the other hand, this design choice may also be considered a strength of the study, since similar investigations previously published in the literature focused on patients with a wide range of ischemic symptoms, thus precluding extrapolation of results to all functional classes.

## CONCLUSIONS

Both aerobic and combined training similarly improved PFWD and HRQoL in PAD patients. There are no advantages to adding strength training to conventional aerobic training. The study does not support the conclusion that combined training is a good strategy for these patients when compared with classic training.
